# Water Soluble Components of ‘Osteocare’ Promote Cell Proliferation, Differentiation, and Matrix Mineralization in Human Osteoblast-Like SaOS-2 Cells

**DOI:** 10.3797/scipharm.1310-25

**Published:** 2014-02-08

**Authors:** Sandeep R. Varma, L. M. Sharath Kumar, Satyakumar Vidyashankar, Pralhad Sadashiv Patki

**Affiliations:** 1 Department of Cell Biology, Research and Development, The Himalaya Drug Company, Bangalore-562 162, India.; 2 Department of Phytochemistry, Research and Development, The Himalaya Drug Company, Bangalore-562 162, India.; 3 Medical Services and Clinical Trials, Research and Development, The Himalaya Drug Company, Bangalore-562 162, India.

**Keywords:** Osteocare, Cell proliferation, ALP activity, OCN, OPN, Col-I, IL-6, Mineralization

## Abstract

Osteocare, a herbal formulation, has been found to be very effective in bone mineralization and support of the microstructure of bone tissue. The water-soluble components of Osteocare (WSCO) induced osteogenic activity in human osteoblast-like SaOS-2 cells. The addition of WSCO (100 μg/ml) to SaOS-2 cells was effective in increasing the cell proliferation by 41.49% and DNA content by 1.9-fold. WSCO increased matrix mineralization in SaOS-2 cells by increased alkaline phosphatase levels and calcium-rich deposits as observed by Alizarin red staining. WSCO markedly increased mRNA expression for osteopontin (OPN), osteocalcin (OCN), type I collagen (Col I) in SaOS-2 cells, and it down-regulated IL-6 mRNA levels in SaOS-2 cells. The present study showed that WSCO plays an important role in osteoblastic bone formation through enhanced activities of ALP, Col I, bone matrix proteins such as OPN and OCN, down-regulation of cytokines like IL-6, as well as promoting mineralization in SaOS-2 cells.

## Introduction

Osteoporosis is a metabolic bone disease characterized by a reduction in bone mass and the microarchitectural deterioration of bone tissue, resulting in skeletal fragility and susceptibility to fractures [1]. The most common type of osteoporosis is the bone loss associated with ovarian hormone deficiency at menopause [2]. During osteoporosis, the total skeletal mass decreases due to an imbalance between the resorption of bone by osteoclasts and subsequent formation of bone by osteoblasts.

Anti-osteoporotic drugs act either by preventing bone resorption (estrogen, calcitonin, biphosphonates, raloxifene) or by agents that stimulate bone formation (anabolic steroids, fluoride). Estrogen replacement therapy is approved for the prevention of bone loss in postmenopausal women and is efficacious in reducing the incidence of skeletal fractures [3]. However, estrogen use and compliance are limited due to its numerous undesirable side effects such as uterine and breast cancer [4]. In addition, the cost of anti-osteoporotic drugs is high to benefit a large population in the developing or even developed countries for the treatment and prevention of osteoporosis [5]. Hence an alternative system for the management of osteoporosis is highly desirable.

Herbal medicine has been widely used to treat bone disease for thousands of years and will continue to be used as a cost-effective alternative to commercial pharmaceutical products [5]. Osteocare is an herbomineral formulation used for the treatment of senile and postmenopausal osteoporosis. Osteocare mainly contains the extracts of *Commiphora mukul* Hook Ex Stock., (Burseraceae), *Alpinia galanga* L., (Zingiberaceae), *Withania somnifera* Dunal., (Solanaceae), and *Terminalia arjuna* W & A., (Combretaceae) that are well-known for their bone mineralization properties [6, 7]. *Terminalia arjuna* is extensively used in the treatment of osteodystrophic conditions [6]. Previous reports on *Terminalia arjuna* showed that the triterpene-saponin fraction of the plant reduces the development of osteoporosis by reducing the bone marrow fat load and by reducing the secretion of pro-inflammatory cytokines [6, 7]. Traditionally, *Commiphora mukul* (Sanskrit Name: Guggulu) is used in the management of fractures and dislocations [6]. Guggulsterone, a steroid present in *Commiphora mukul* inhibits osteoclastogenesis induced by the receptor activator of NF kappa B ligand [8]. The therapeutic use of *Withania somnifera* for bone weakness in traditional medicine was reported [9, 10]. The estrogen-like withanolides present in *Withania somnifera* confers the anti-osteoporotic potential to this plant [11]. *Alpinia galanga* is widely used as a part of normal dietary intakes as well as in the traditional system of medicine viz., Ayurveda, Unani, Chinese, and Thai folk medicine [12]. In the Unani system of medicine, the rhizome of this plant is used as a cure for bone weakness and healing fractures [13]. The constituent plants of Osteocare were identified and certified by a botanist and the voucher specimen of each constituent plant has been archived in the herbarium of Research and Development Centre, The Himalaya Drug Company, Bangalore, India. The composition of Osteocare with respect to the scientific names of the plants, parts used, drug extract ratio, and solvent used is given in Table 1.

The therapeutic effects of Osteocare on osteoporosis and bone loss were reported by many workers [2, 14–16]. However, the cellular and molecular mechanisms of Osteocare and its effects on proliferation, differentiation, and matrix mineralization have yet to be explored.

Established osteoblast-like cell lines are particularly useful models to study signalling pathways in response to stimulation by osteotropic factors. SaOS-2 cells have been used to assess the effects of herbal compounds on the proliferation, differentiation, and matrix mineralization of osteoblastic cells [17–21]. The present study is aimed to delineate the effects of Osteocare on the proliferation, differentiation, and matrix mineralization of human osteoblastic SaOS-2 cells.

## Results

### Effect of WSCO on Viability and Cell Proliferation

**Fig. 1:**
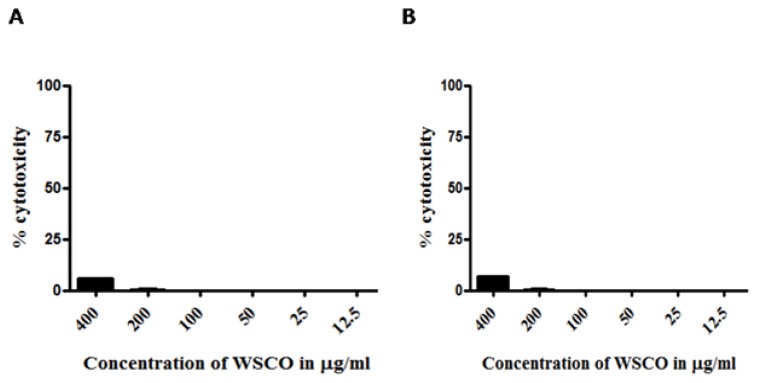
Cytotoxicity of WSCO on SaOS-2 cells. SaOS-2 cells were incubated for 48 and 72 h with different concentrations of WSCO and the cell viability was determined using the MTT assay. (A) Cytotoxicity after 48 h (B) Cytotoxicity after 72 h. Data are expressed as percentage of control. Results are shown as ± SD of the three experiments.

WSCO showed no cytotoxic effects on SaOS-2 cells after 48 and 72 h at the test doses (Fig. 1A, B). Non-toxic concentrations of WSCO were taken for further experimentation. A stimulatory effect on osteoblastic proliferation was observed when the cells were treated with WSCO and the maximum stimulation was observed at 100 μg/ml after 48 h (Table 2). 17β-estradiol showed increased cell proliferation with 80.68 and 77.64% at 48 and 72 h, respectively. WSCO at 100 μg/ml increased the DNA yield by 1.9 fold, whereas at 50 and 25 μg/ml, WSCO increased the DNA yields by 1.6- and 1.4-fold, respectively (Table 2).

### Effect of WSCO on ALP Activity

**Fig. 2:**
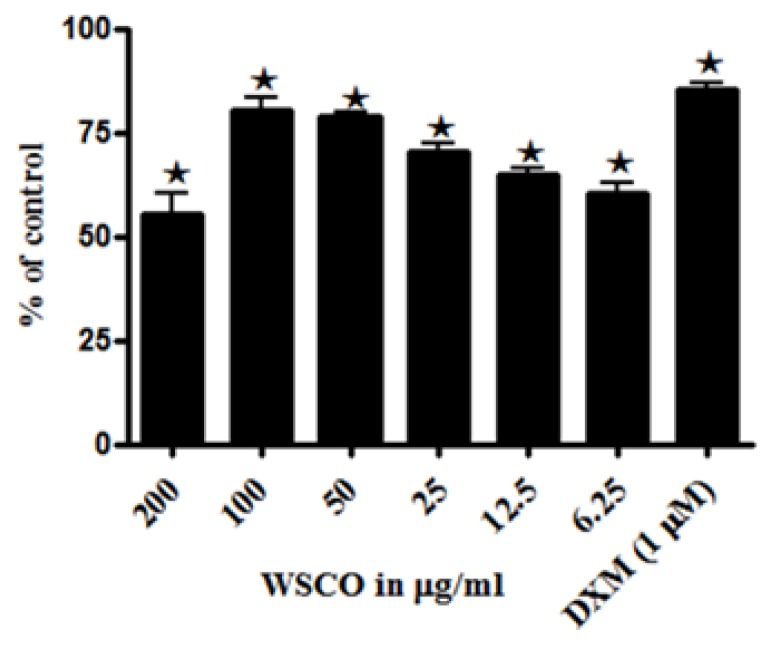
Effects of WSCO on ALP activity in SaOS-2 cells. SaOS-2 cells were incubated for 48 hr with different concentrations of WSCO and the ALP activity was determined. Data are expressed as the percentage of control. Results are shown as ± SD of the four experiments.

**Fig. 3:**
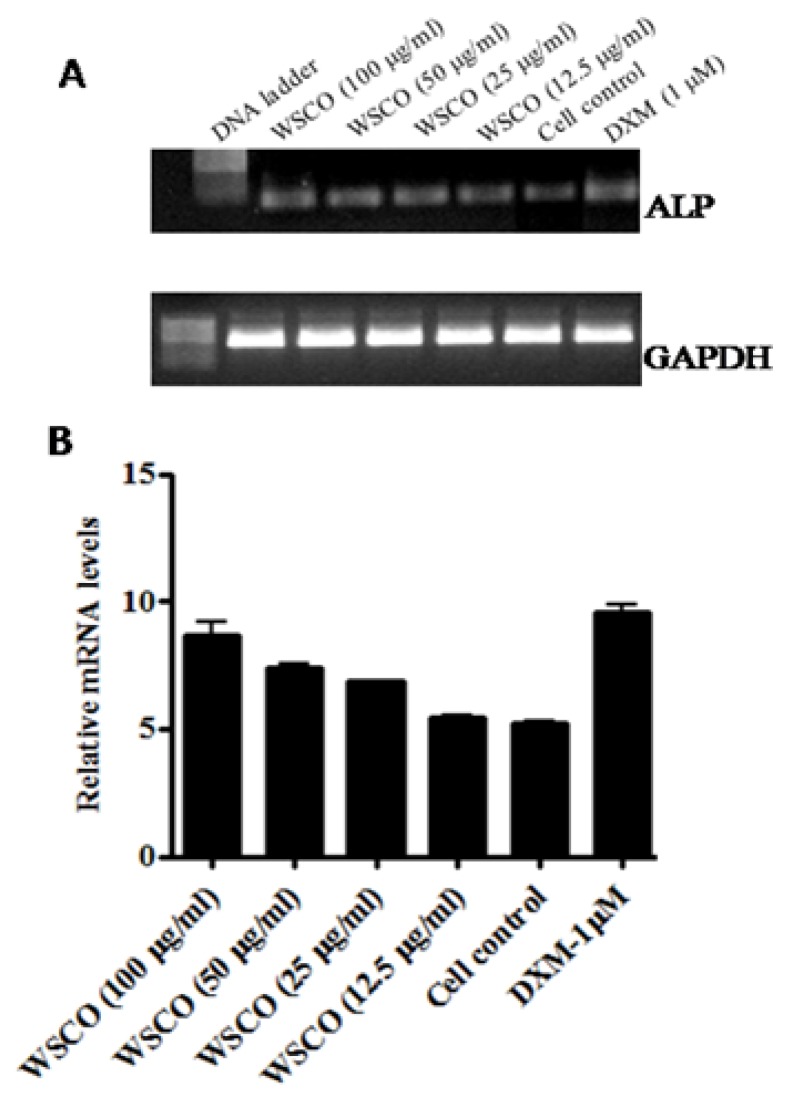
Effect of WSCO on ALP gene expression in SaOS-2 cells. (A) RT-PCR profile of ALP and GAPDH. (B) Densitometric analysis of gene transcripts. Data is expressed as mean (±SE).

WSCO showed increased ALP activity in SaOS-2 cells over 48 h, and the maximal effect was reached when the cells were treated with 100 μg/ml WSCO (Fig. 2). ALP activity started declining at concentrations below 50 μg/ml. The activity of ALP was found to be at the maximum at 100 and 50 μg/ml and the proliferation was also found to be at the maximum at these concentrations. Thus, these doses were found to be effective and further studies were carried out using these doses. Dexamethasone enhanced ALP activity in SaOS-2 cells. The increase in the ALP activity by WSCO at 100 μg/ml was comparable to that of dexamethasone. The increase in the ALP activity was further confirmed by RT-PCR analysis. The mRNA expression of ALP was increased significantly in cells treated with WSCO when compared to untreated cells (Fig. 3).

### Effect of WSCO on Extracellular Matrix Gene Expression

**Fig. 4:**
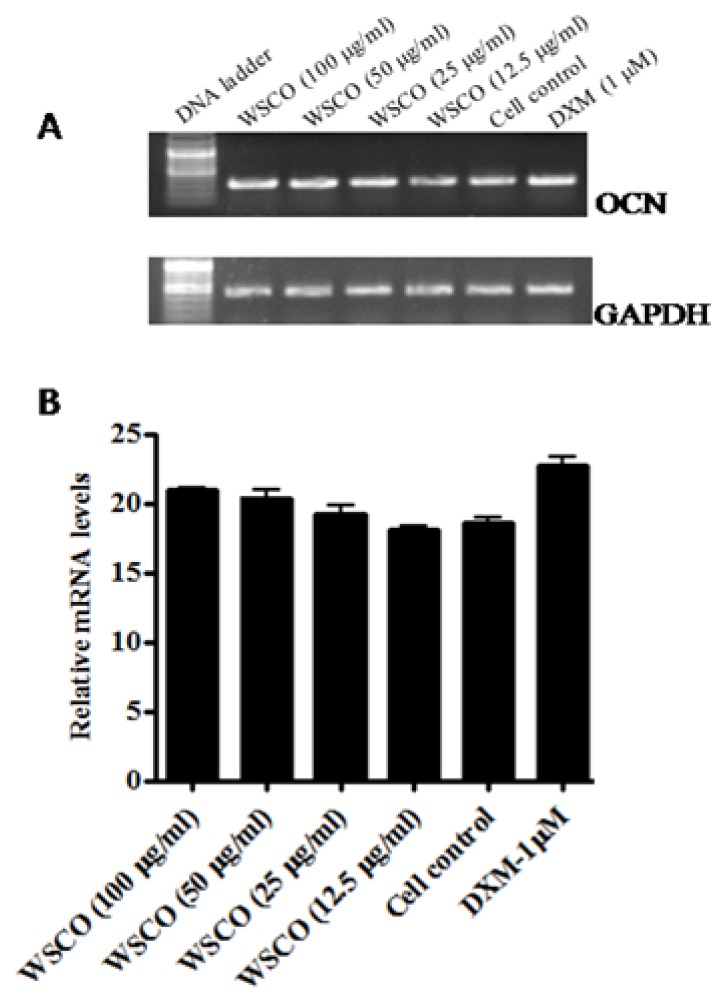
Effect of WSCO on OCN gene expression in SaOS-2 cells. A) RT-PCR profile of OCN and GAPDH. (B) Densitometric analysis of gene transcripts. Data is expressed as mean (±SE).

**Fig. 5:**
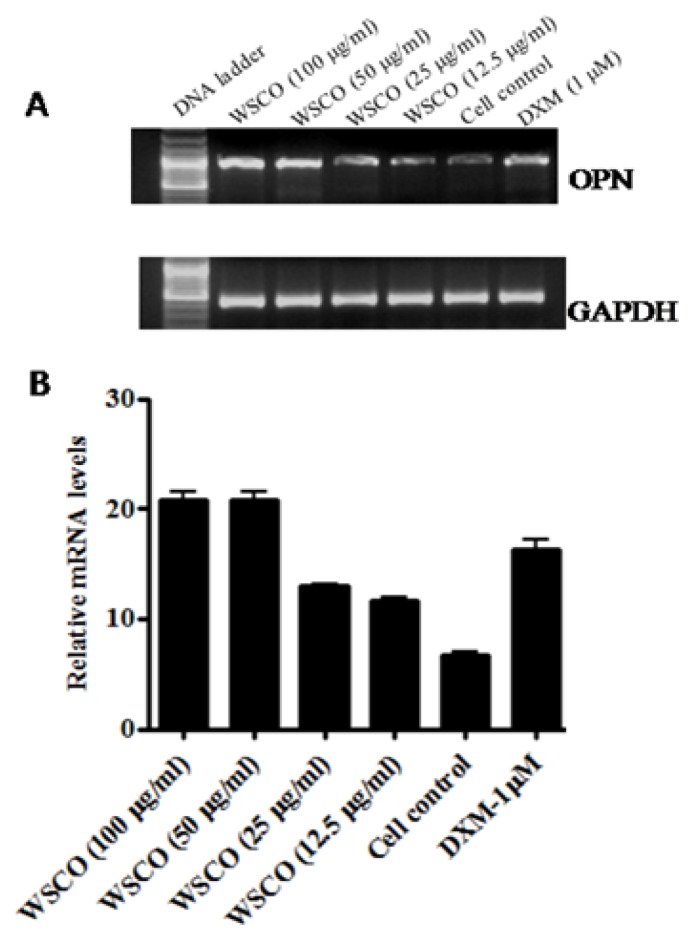
Effect of WSCO on OPN gene expression in SaOS-2 cells. (A) RT-PCR profile of OPN and GAPDH. (B) Densitometric analysis of gene transcripts. Data is expressed as mean (±SE).

**Fig. 6:**
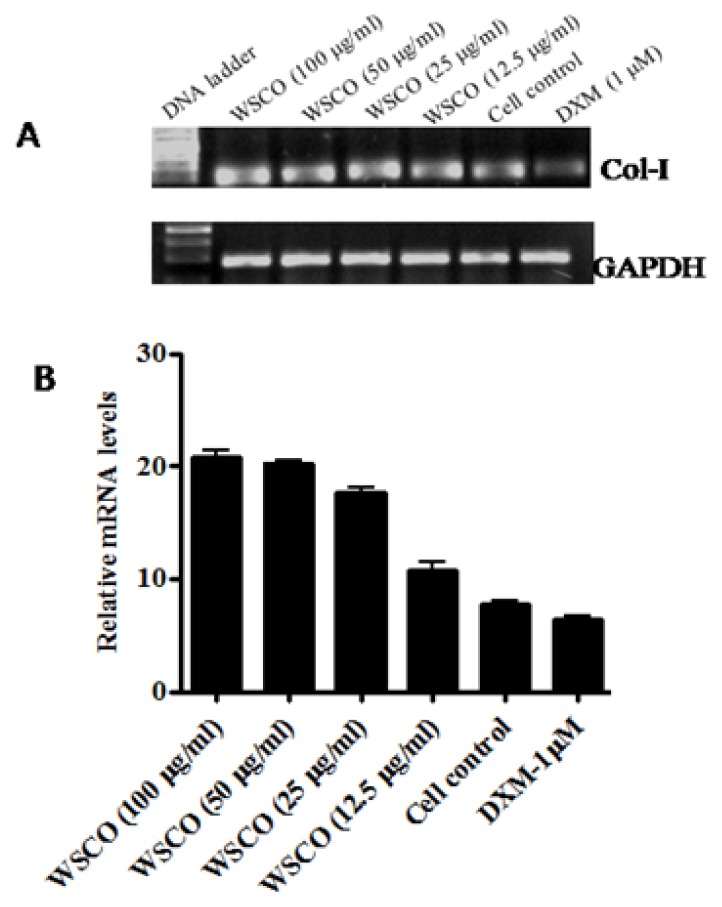
Effect of WSCO on Collagen I gene expression in SaOS-2 cells. A) RT-PCR profile of Col I and GAPDH. (B) Densitometric analysis of gene transcripts. Data is expressed as mean (±SE).

**Fig. 7:**
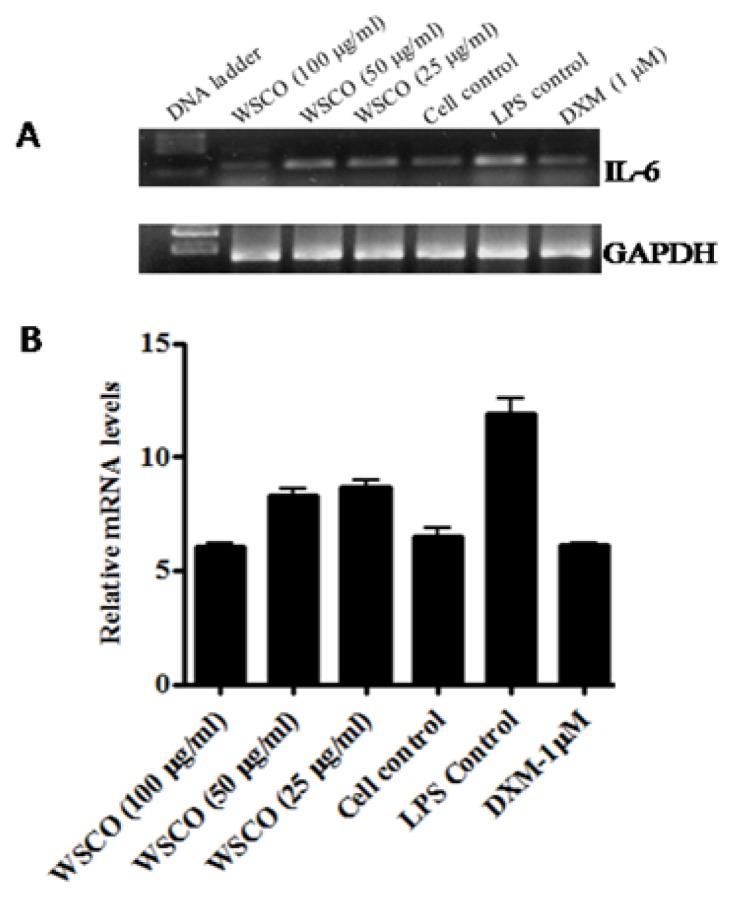
Effect of WSCO on LPS-induced IL-6 gene expression in SaOS-2 cells. (A) RT-PCR profile of IL-6 and GAPDH. (B) Densitometric analysis of gene transcripts. Data is expressed as mean (±SE).

As the expression of OCN, OPN, and Col I changes during the maturation and differentiation of osteoblasts, the effects of WSCO on the expression of these genes were examined. RT-PCR analysis showed a dose-dependent increase in the mRNA expression of OCN, OPN, and Col I genes compared to the control cells (Fig. 4–6). However, WSCO inhibited LPS-induced IL-6 gene expression in SaOS-2 cells (Fig. 7). Dexamethasone up-regulated the gene expression for ALP, OCN, OPN, and IL-6, whereas Col I expression was down-regulated by dexamethasone (Fig. 4–7). The internal control, GAPDH was uniformly amplified in all the samples.

### Effect of WSCO on Mineralization

**Fig. 8:**
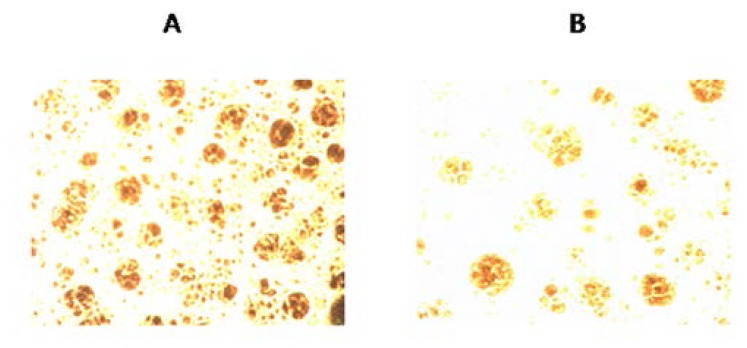
Effects of WSCO on mineralization in SaOS-2 cells. Cells were treated with WSCO for 14 days, formalin-fixed, stained with alizarin red, and observed under the microscope and photographed (magnification × 40). (A) Cells treated with WSCO at 100 μg/ml. (B) Cell control.

The effect of WSCO on matrix mineralization was assessed in SaOS-2 cells at concentrations of 100, 50, and 25 μg/ml. The mineralized nodule formation in SaOS-2 cells cultured for 14 days with WSCO is shown in Fig. 8. The mineralized nodules appeared as red patches depending on mineral deposition. Maximum mineralization was observed with 100 μg/ml WSCO, whereas at 50 and 25 μg/ml, the extent of mineralization was less as compared to higher concentrations.

## Discussion

The promotion of osteoblast activity with drugs that lack cytotoxicity can be a better approach for drug development to treat osteoporosis [21]. The present study demonstrated that WSCO potentially induced osteoblastic differentiation markers such as ALP, OPN, OCN, Col I, and mineralization in SaOS-2 cells. This is the first report to show that WSCO regulates much of the tightly linked control between the maturation and differentiation in SaOS-2 cells through the increased synthesis of matrix proteins, which ultimately stimulates mineralization.

Initially, the putative cytotoxic effect of WSCO on SaOS-2 cells was checked since many therapeutic agents have been shown to possess severe side effects [21]. WSCO had no effect on the viability of the SaOS-2 cells. The results of cell proliferation were comparable to that of 17β-estradiol, used as the standard in cell proliferation experiments [25]. The osteogenic activity of WSCO was confirmed by the MTT assay, showing increased DNA content in SaOS-2 cells.

ALP is the most widely recognised biochemical marker for osteoblastic activity [26, 27]. Though the precise mechanism of action is poorly understood, the enzyme is believed to play an important role in bone metabolism [28, 29]. WSCO at concentrations of 100 and 50 μg/ml exhibited significant, positive effects on ALP activity. These results were comparable to that of dexamethasone, used as a standard in the present study [30]. The increase in the ALP activity was further confirmed by RT-PCR by employing specific primers and the results showed a dose-dependent increase in the ALP gene expression as compared to the control. Thus, it could be concluded that WSCO stimulated osteoblastic activity at least in part by enhancing the synthesis of ALP. In addition to these *in vitro* observations, the positive effects of Osteocare on ALP activity have also been reported during clinical trials of rickets in rats [14]. Taken together, the increase in ALP activity due to WSCO supports the earlier views on the anabolic effects of Osteocare on bone formation [2, 15, 16].

The formation of bone involves a complex series of events that include the proliferation of osteoblasts and their differentiation, eventually resulting in the formation of a mineralized extracellular matrix. A number of genes including alkaline phosphatase, type I collagen, and osteocalcin are highly expressed in the differentiation period [31]. Osteocalcin is a late marker of osteoblastic differentiation that is closely related to osteoblastic maturation and matrix mineralization [32–34]. Data from osteocalcin-deficient mice suggest that osteocalcin may limit *in vivo* bone formation [35]. The present study demonstrated a dose-dependent increase in the levels of osteocalcin by WSCO after 14 days of the SaOS-2 culture. The effects of WSCO in enhancing OCN gene expression approached those of dexamethasone – a glucocorticosteroid that stimulates OCN formation in osteoblastic cells [30, 36].

Osteopontin is a bone matrix protein secreted by osteoblasts, and is regarded as the last in a chronological sequence of markers of osteoblastic differentiation. OPN expression is enhanced by hormones and cytokines which regulate mineral growth *in vitro* and *in vivo* [37]. WSCO dose-dependently increased OPN mRNA expression after 14 days of being cultured. Osteoblasts abundantly synthesize and secrete collagen I, a major bone matrix constituent and extracellular macromolecule in osteoblast cultures. Osteoporosis leads to the reduction in the collagen content which in turn leads to finer fibrils, modified crosslinks, and reduced calcification which ultimately leads to the fragility of the bone [38]. In the present study, SaOS-2 cells showed increased Col I gene expression upon treatment with WSCO at various doses after 14 days of being cultured. However, dexamethasone suppressed the Col I mRNA in SaOS-2 cells, as reported earlier [30, 39].

IL-6 is a pluripotent cytokine and is reported to inhibit osteoblast differentiation, thus inhibiting bone formation [40, 41]. Since the gene expression levels of IL-6 was weak, LPS was used to stimulate the IL-6 gene expression and the effect of WSCO on LPS-stimulated cells was observed. WSCO inhibited LPS-induced IL-6 gene expression in SaOS-2 cells. This observation confirmed the osteoblastic stimulating properties of WSCO.

Finally, the present study showed the effect of WSCO on mineralized nodule formation after 14 days of the SaOS-2 culture. WSCO at 100 μg/ml displayed marked mineral-positive Alizarin red staining. Alizarin red staining is particularly useful for the assessment of the osteogenic properties of drugs by evaluating the calcium-rich deposits in a culture [42]. This result shows that WSCO promoted matrix mineralization *in vitro*, through increased synthesis and secretion of matrix proteins.

In conclusion, our study showed that WSCO promoted the osteogenic activity by cell proliferation and stimulated ALP levels in SaOS-2 cells. WSCO stimulated the osteogenic proteins, osteopontin and osteocalcin, up-regulated Collagen I, inhibited IL-6 mRNA, and increased matrix mineralization in SaOS-2 cells. The results obtained from the present *in vitro* study provide strong evidence supporting the anti-osteoporotic properties of this drug.

## Experimental

### Chemicals

McCoy’s medium, fetal bovine serum, 3-(4,5-dimethylthiazol-2-yl)-2,5-diphenyl tetrazolium bromide (MTT), 17β-estradiol, alizarin red, para-nitrophenyl phosphate, diethanolamine, ascorbic acid, TRI reagent, custom-prepared oligonucleotides, lipopolysaccharide (LPS), and dexamethasone were obtained from Sigma (St. Louis, MO, USA). Penicillin, streptomycin, and magnesium chloride were from Hi-media (Mumbai, India). MMLV reverse transcriptase, deoxynucleotide triphosphates, and Taq DNA polymerase were purchased from MBI Fermentas (Amherst, NY, USA). All other reagents and chemicals used were of molecular biology grade.

### Sample Preparation

Uncoated Osteocare granules were obtained from the Formulation and Development department of Himalaya Drug Company. The herbomineral formulation was pulverised using a mortar and pestle and sieved with a mesh (0.5 μm) to obtain a fine powder. This powder was then weighed and extracted with a 1:4 volume of powder: water at room temperature for 24 h. The water-soluble contents of Osteocare (WSCO) were extracted by centrifugation at 5000 g for 30 min. After centrifugation, the supernatant was collected, lyophilized, and used in all the experiments.

### Liquid Chromatography-Mass Spectrometer Analysis

The LC-MS/MS instrument consisted of an HPLC (Shimadzu LC-20AD) coupled with API-2000 mass spectrometer-MS/MS, [Applied Biosystem/MDS SCIEX, Canada]. 20 μl WSCO extract was prepared at a concentration of 2 mg/ml in water (made up to the volume with methanol) and injected through the SIL-HTC Shimadzu autosampler for phytochemical screening. The separation of WSCO extract was achieved through the Luna RP-C_18_ (5μM, 250 × 4.6mm) (Phenomenex Torrance, CA, USA) column. The sample (20 μl of WSCO test solution) was injected through the SIL-HTC Shimadzu autosampler and the column oven temperature was maintained at 40°C throughout the analysis by the CTO-10ASVP column oven.

The separation and identification of Withanolide–A, Withanoside–IV, and Withanoside–V were achieved by a mobile phase consisting of water (Merck, maker) with 0.1% formic acid in pump A and acetonitrile (Merck, maker) with 0.1% formic acid in pump B. A linear gradient program was: 0–20 min of 5% to 75% of B (binary), 20–25 min of 75% to 5% B (binary), 25–30 min of 5% B (linear) delivered at a flow rate of 1 ml/min with a splitter and the run time was about 30 min.

The separation and identification of Guggulsterone *E* & *Z* and arjunolic acid were achieved by a mobile phase which was the combination of A (0.1% formic acid in water) and B (acetonitrile with 0.1% formic acid). A binary gradient ratio A: B (30:70) was delivered at a flow rate of 1 ml per min with a splitter and the run time was about 20 min.

An API-2000 mass spectrometer coupled with an electron spray ionization (ESI) interface was used to obtain the MS/MS spectrum. Batch acquisition and data processing were controlled by Analyst 1.5 version software. The ionization conditions were optimized and the following conditions were adopted – Curtain gas (CUR) 25 psi; Focusing potential (FP) (±) 400 V; Entrance potential (EP) (±) 10 V; Ionisation Source Temperature 420°C; Ion source Gas 1 (GS1) 55 psi; and Ion source Gas 2 (GS2) 65 psi. The collision energy (CE) for MRM of the precursor to the product ion was optimized by a multiple run through LC until the most intense precursor to the product ion transition state was obtained. The data was recorded in negative and positive multiple reaction monitoring (MRM) mode.

### Culture Conditions

Human osteoblast-like SaOS-2 cells were procured from the National Centre for Cell Sciences (NCCS) Pune, India. The cells were grown in McCoy’s medium supplemented with 10% FBS, 100 IU/ml penicillin, and 100 mU/ml streptomycin in a humidified incubator at 37°C and 5% CO_2_.

### Cytotoxicity

The colorimetric MTT assay was done to determine whether any of the test concentrations of WSCO were toxic to the SaOS-2 cells. For this, cells with a seeding density of 1 × 10^4^ cells per ml were seeded in two 96-well flat bottom culture plates and incubated overnight. The cells were exposed to different concentrations of WSCO diluted in McCoy’s medium containing 2% FBS and incubated for 48 and 72 h, and the MTT assay was carried out. The percentage toxicity was calculated over the control. The non-toxic concentrations of WSCO were used for further experiments.

### Cell Proliferation

SaOS-2 cells were harvested by trypsinisation and resuspended in serum-free McCoy’s medium. They were plated at an initial seeding density of 1 ×10^4^ cells per well in a volume of 100 μl per well in two 96-well microplates. WSCO at different concentrations were added to the wells. 17β-estradiol was used as the standard drug for the cell proliferation experiments in the present study. 17β-estradiol at 1 nM was added to separate the wells and the cell control was also maintained. Cell proliferation was measured by the MTT assay.

To estimate the total DNA content in the treated/untreated cells, SaOS-2 cells were plated in 40 mm Petri plates with an initial seeding density of 1 × 10^5^ cells per ml in serum-free media and incubated with/without the drug samples in a humidified atmosphere of 5% CO_2_ at 37°C for 48 h. The total DNA was extracted from the treated/untreated cells using the TRI reagent and the DNA was quantified using a spectrophotometer at 260 and 280 nm and the total yield/number of cells were calculated. The total DNA yield with different doses of WSCO was compared with the untreated cells and the fold increase was calculated.

### Assay of ALP Activity

The quantitative alkaline phosphatase activity of the culture was determined by an assay based on the hydrolysis of *p*-nitrophenyl phosphate to p-nitrophenol based on the protocol of Zhang et al. [22]. Dexamethasone (1 μM) was used as a standard in this experiment. A standard curve was prepared with *p*-nitrophenol. Each value was normalized to the protein concentration.

### Analysis of mRNA Expression

SaOS-2 cells were seeded in 40 mm culture dishes (1 × 10^5^ cells per ml) and incubated overnight. Cells were treated with WSCO at 100, 50, 25, and 12.5 μg/ml. Dexamethasone (1 μM) was used as a standard in all the gene expression experiments. Cell control was also maintained. Treated and untreated cells were incubated for 14 days at 37°C. For the ALP and IL-6 gene amplifications, the incubation period was restricted to 48 h. For IL-6 gene amplification, SaOS-2 cells were induced with LPS (1 μg/ml) for one hour followed by the addition of WSCO at different concentrations and further incubation for 47 h at 37°C. RNA isolation and RT-PCR was carried out as described by us earlier [23]. PCR amplification was carried out to amplify the genes encoding ALP, osteocalcin, osteopontin, Collagen I, and IL-6 using species-specific primers (Table 3). GAPDH was used as an internal control for all the experiments. The three-step PCR cycles consisted of denaturation at 94°C for 1.5 min, annealing at a specific temperature for 1.5 min, and extension at 72°C for 1.5 min (Table 1). The PCR amplification was carried out for up to 30 cycles and the final extension was performed at 72°C for 10 min. The amplified products were resolved in a standard 1.5% agarose gel stained with ethidium bromide and photographed under UV light. The gel was subjected to densitometric scanning and the band intensity of the cDNA fragment of each gene of interest was normalized against the band intensity of the cDNA fragment of the house keeping gene, GAPDH using Image J software (Rasband, USA).

### Matrix Mineralization

The formation of mineralized nodules was analyzed using the Alizarin red staining method for calcium deposition [24]. Briefly, SaOS-2 cells were cultured in 40 mm petriplates and treated with different dilutions of WSCO. Cell control was also maintained. The cultures were incubated at 37°C with 5% CO_2_ for 14 days. At the end of the incubation period, the media was removed by inversion and the plates were fixed with 10% formalin for 10–15 min, followed by washing several times with distilled water. The Alizarin red stain was added to the plates and incubated for 15 min and washed with distilled water to remove the excess amount of the dye. The plates were then observed under a fluorescent microscope (40 x) and the photographs were taken and recorded. The mineralized nodules were labelled as red spots.

### Statistical Analysis

Statistical analysis of the data was carried out using the GraphPad Prism4. Unpaired Student’s *t* - test was used to analyze the data. Data are represented as mean ± SEM and P <0.05 was considered significant.

## Figures and Tables

**Tab. 1:** Composition of Osteocare granules

No	Ingredients	Part used	Quantity used (mg)	Drug extract ratio	Solvent used
1	*Commiphora mukul*	Oleo-gum-resin	32.8	1.89:1	water
2	*Alpinia galanga*	rhizome	7.6	7.69:1	water
3	*Terminalia arjuna*	bark	6.4	5:1	water
4	*Withania somnifera*	root	6.4	6.66:1	water
5	Kukkutandtvak bhasma*	–	520	–	–
6	Godanti bhasma#	–	170	–	–

* hen’s egg shell calx;

# gypsum calx.

**Tab. 2:** Cell proliferative activity of WSCO in SaOS-2 cells

Sample	Concentration	% cell proliferation		DNA yield μg /ml	DNA fold increase (over control)

		48 h	72 h		
	200 μg /ml	23.32	18.01	5.85	1.3
	100 μg /ml	41.49	26.21	8.55	1.9
WSCO	50 μg /ml	36.05	21.24	7.20	1.6
	25 μg /ml	28.74	18.04	6.30	1.4
	12.5 μg /ml	21.18	10.87	5.90	1.3
17β-Estradiol	1 nM	80.68	77.64	10.05	2.2
Cell control	–	–	–	4.50	–

**Tab. 3:** List of primer sequences used in the study

Gene	Forward primer (5′-3′)	Reverse primer (5′-3′)	Anneal. temp.	Size
ALP	ACCTCGTTGACACCTGGAAG	CCACCATCTCGGAGAGTGAC	55°C	189
OPN	CAGCCATGAATTTCACAGCC	GGGAGTTTCCATGAAGCCAC	60°C	307
OCN	CATGAGAGCCCTCACA	AGAGCGACACCCTAGAC	55°C	310
Col I	TCTTGGTCGGTGGGTGACTCT	CCCCCTCCCCAGCCACAAAG	44°C	360
IL-6	GTACCCCCAGGAGAAGATTC	CAAACTGCATAGCCACTTTC	60°C	819
GAPDH	ACCACAGTCCATGCCATCAC	TCCACCACCCTGTTGCTGTA	60°C	453
